# “Philosophysics” at the University of Vienna: The (Pre-)History of Foundations of Quantum Physics in the Viennese Cultural Context

**DOI:** 10.1007/s00016-022-00290-y

**Published:** 2022-10-17

**Authors:** Flavio Del Santo, Emanuel Schwarzhans

**Affiliations:** Institute for Quantum Optics and Quantum Information, Austrian Academy of Sciences, Boltzmanngasse 3, 1090 Vienna, Austria

**Keywords:** History of modern foundations of quantum mechanics, history of physics in Vienna, physics and philosophy, postwar physics, oral history

## Abstract

Vienna today is one of the capitals of research on foundations of quantum physics. We reconstruct the development of modern physics in Vienna, with a focus on foundations of quantum mechanics (FQM), which is a sub-field of quantum mechanics. We show that the influence of Erwin Schrödinger and, in more recent years, the initiatives of Anton Zeilinger—the two main intuitive reasons—cannot alone account for today’s outstanding research landscape on FQM in Vienna. We highlight four additional factors that played a crucial role in the development of foundational research in Vienna: 1) the Viennese heritage of the cultural golden age just before World War II; 2) the long-lasting institutional connection between the faculty of physics and philosophy; 3) a rise of several initiatives that gave forum to the interplay of physics and philosophy; and 4) the influence of several external scholars in the Viennese scientific landscape. Our analysis is informed by interviews with the most pertinent scientists, a detailed survey of the relevant social networks, and review of the main primary literature.

## Introduction

Vienna is today recognized as a major center for the studies on FQM. This field of research is unconventional within physics, for it often addresses questions that border on philosophical investigation. Only recently it has become part of mainstream physics, largely by the virtue of practical applications such as secure quantum communication and the promise of a universal quantum computer.

Our aim is to study the historical developments that made it possible for FQM to flourish in Vienna. More specifically, we will address the following question: was there something exceptional in the academic and, more generally, the cultural landscape that made of Vienna a particularly fertile ground for FQM compared with other places?

At first sight, the answer seems straightforward. First, many preeminent physicists concerned with FQM studied and worked in Vienna, most notably one of the founding fathers of quantum theory, Erwin Schrödinger (1887–1961). Second, in the past few decades, the seminal initiatives of Anton Zeilinger (b. 1945) have deeply shaped physics research at Austrian institutions. Yet, this twofold explanation is not completely satisfactory; as we shall see, Schrödinger’s influence cannot be held accountable for the developments of the research on FQM in Vienna, except potentially indirectly due to his renown. Moreover, although it is unquestionable that Zeilinger—who indeed made pivotal contributions to FQM—was the driving force for the establishment of the research on FQM in Vienna, it is more than doubtful that this could have happened with the same success anywhere else. In fact, if we distance ourselves from the hagiographic narrative, we should realize that the cultural context in which scientists live and work plays a fundamental role in their intellectual formation, for the development of novel ideas and for the acceptance and establishment thereof. In the case of Zeilinger, historian of physics Olival Freire’s words reflect this idea: “Zeilinger’s intellectual style is marked by a deep curiosity, which was directed towards science during his undergraduate studies and favored by the flexible curriculum at University of Vienna at that time. In addition, he benefited from Rauch’s support to research on foundations of quantum mechanics and from the intellectual climate of physics in Vienna—with its mix of science and philosophy—a legacy coming from the late nineteenth and twentieth centuries.”^1^

We will show that the acceptance of FQM in Vienna was indeed the result of a combination of cultural and institutional factors that provided a fertile ground, together with the rise of several initiatives that gave forum to the interplay of physics and philosophy as well as the influence of several external scholars.

Indeed, the role that Vienna played at the turn of the twentieth century as a major multicultural center for art, philosophy, and science was reflected also in a flourishing landscape of intellectual debates at the boundary between physics and philosophy, such as the Boltzmann–Mach controversy, or the Popper–Vienna Circle dispute about scientific method. Despite the fact that this exceptional period of intellectual blossoming was shattered by the advent of fascism and the outbreak of World War II, its legacy continued to influence the Viennese scientific landscape throughout the second half of the century.

With the transition into Cold War, a new scientific paradigm took hold. In addition to an increased focus on military research, a hyper-pragmatic attitude emerged, mostly devoid of any fundamental and philosophical concerns. Such an attitude was epitomized by the expression “shut up and calculate!”^2^ Following the United States as the leading Western scientific force, many European countries adhered to such an attitude. Research became dominated by particle physics, where research projects turned into collective enterprises that involved tens if not hundreds or thousands of physicists (so-called big science). At the same time, research on FQM virtually disappeared from scientific programs, and was regarded with suspicion or actively opposed.

Also in Austria, and in particular in Vienna, a large part of the physics that was rebuilt in the postwar period focused on particle physics and was *prima facie* conducted within the standard pragmatism of the time. However, in Vienna this drastic separation between science and its philosophical and fundamental aspects was never as complete as almost everywhere else. First, at the institutional level, at the University of Vienna, until as late as 1975 physics was a department of the Faculty of Philosophy.^3^ The final exam (called *Rigorosum*) to become *Dr. Phil.* in physics included philosophy as a mandatory subject. Consequently, Viennese physics students received some formal education in philosophy. Second, although most of the teaching and research activities in Viennese universities were more standardized than in other cities and countries, many leading physicists were actively involved in a number of initiatives that were rooted in a genuine interest in philosophy of science and foundational research. Indeed, as Reinhold Bertlmann (b. 1945)—an Austrian physicist who closely collaborated with John S. Bell (1928–90) and, together with Zeilinger, was among the first to introduce FQM into teaching at the University of Vienna in the early 1990s—put it, “officially they just followed this rule ‘shut up and calculate,’ but in their heart they were open and unofficially they discussed [foundational problems]…. There was still an atmosphere of philosophy.”^4^ It should be remarked that, contrary to other cases, such as Italy or the United States^5^—where interest in foundations was revived between 1970 and 1980s, motivated by political and ideological reasons, after a period of dormancy—in Vienna, philosophy and physics always maintained a relatively strong bond with a certain continuity. According to our account, this happened primarily for conservative reasons, namely for the strength that this bond had acquired in fin-de-siècle Vienna. Indeed, Vienna has a reputation for its notorious cultural conservatism, as crystallized by the famous dictum attributed to the Austrian composer Gustav Mahler: “If the world ends, I would go to Vienna; there everything happens fifty years later.”

This tradition of philosophical interest and fundamental debate in physics in the Austrian capital was kept alive and recast by several physicists, such as theoreticians Herbert Pietschmann (b. 1936) and Roman U. Sexl (1939–86), and the experimentalist Helmut Rauch (1939–2019). In particular, Pietschmann and the philosopher Gerhard Schwarz (b. 1937) founded, in 1964, a “Philosophical-Scientific Working Group” (*Philosophisch-Naturwissenschaftlicher Arbeitskreis*), which catalyzed the curiosity of physicists toward philosophical issues and allowed a structured forum for debate among physicists and philosophers.^6^

**Fig. 1:**
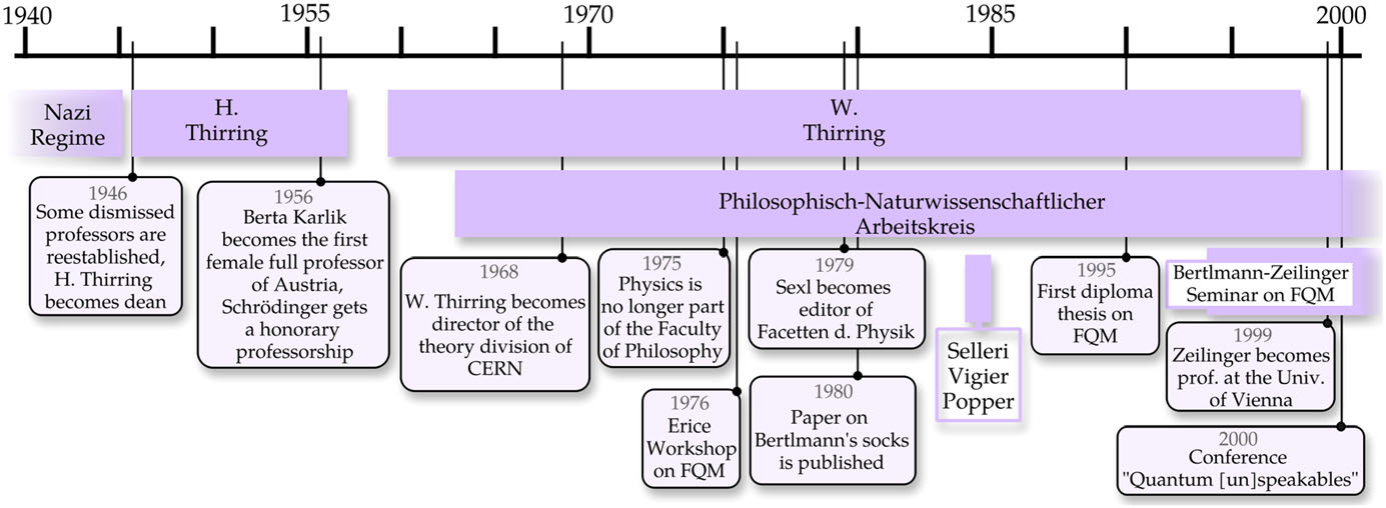
Timeline of selected events on matters of physics (and philosophy) in Vienna as recollected in the main text. The purple strips represent extended periods

Furthermore, as anticipated, despite the possibility that the rise of foundational research could be greatly ascribed to the influence of Schrödinger—who came back to Vienna in his late years (since 1956)—we document that he deliberately did not establish a school, and did not essentially contribute further to FQM while in Vienna. Conversely, a series of external influences helped pave the way for the flourishing research environment on FQM in Vienna. This was the case for Franco Selleri (1936–2013)—the initiator of the revival of FQM in Italy^7^—who had regular interactions with the Viennese community and even spent a sabbatical in Vienna where he lectured on FQM.^8^ Also, John S. Bell—who revolutionized the field of FQM more than anyone else in the postwar era by formulating the inequalities that bear his name^9^—gave occasional talks in Vienna on the issue of local realism and he was the single major intellectual influence on Bertlmann (figure 1).

Our analysis is mostly based on interviews with significant historical figures, a detailed examination of relevant social networks, and a review of the primary literature. This allows us to access information that is not available in written sources, including the perception of the general atmosphere, in particular regarding the local acceptance of foundational and philosophical questions.

## Physics (and Philosophy) in Vienna before World War II

Vienna has an outstanding physics tradition, which dates back to the second half of the nineteenth century when the city experienced an intellectual golden age. In those years, the University of Vienna had among its professors physicists like Christian Andreas Doppler (1803–53), Josef Loschmidt (1821–95), Joseph Stefan (1835–93), Ernst Mach (1838–1916), Ludwig Boltzmann (1844–1906), and Franz Serafin Exner (1849–1926), who collectively laid the groundwork for an exceptionally prolific physics milieu in the following years. The three Austrian Nobel Laureates in physics, Erwin Schrödinger, Victor Francis Hess (1883–1964), and Wolfgang Pauli (1900–1958), as well as Lise Meitner (1878–1968) and Paul Ehrenfest (1880–1933), were all academic children of that extraordinary period.

In particular, the intellectual debate between Mach’s empiricism and Boltzmann’s realism had a lasting impact on foundations of physics and general philosophy of science, and gave momentum to the interplay between physics and philosophy. Notably, despite his physics background, Mach was appointed the world’s first chair in history and philosophy of science.^10^

**Fig. 2:**
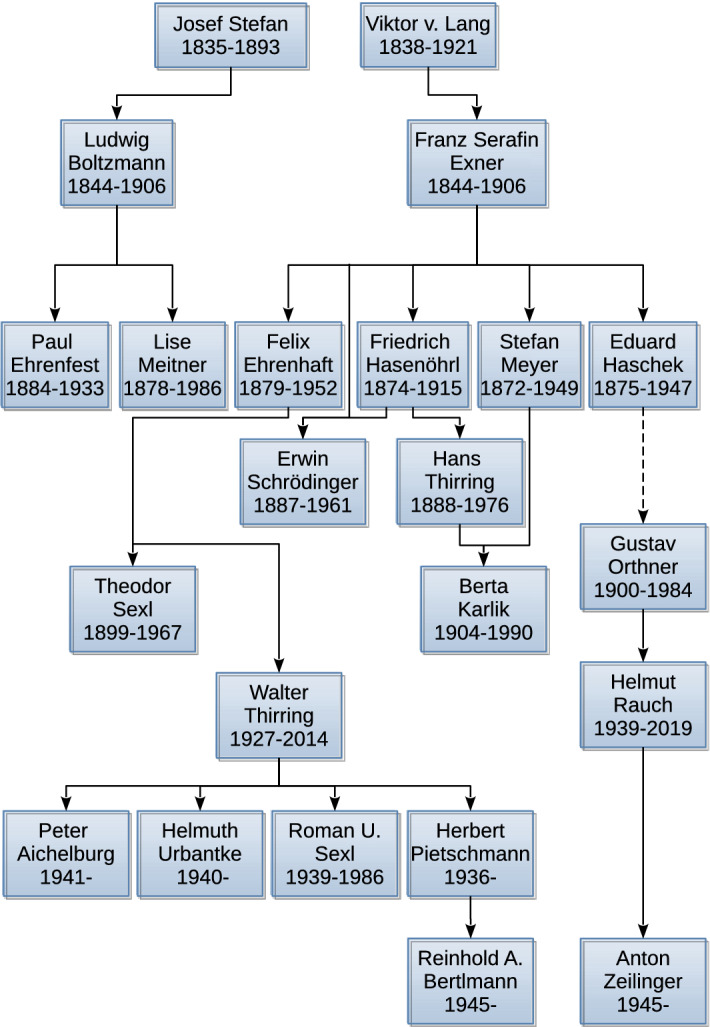
Academic “family tree” of the relevant personalities mentioned in this work. The links between them indicate a supervisor-doctoral student relation (or in the rare cases in which it was not possible to certify this, an acknowledged major influence). The link between Eduard Haschek and Gustav Ortner is dotted because we have inconclusive evidence as to whether there was a strong connection between them

The interplay between physics and philosophy was continued notably by two outstanding intellectual circles: The “Exner Circle” and the “Vienna Circle,” which both exerted lasting influence on the scientific community in Vienna.^11^ In fact, Exner anticipated the rise of fundamental indeterminism before quantum mechanics. He furthermore formed a school of physicists who were to become highly influential figures in Vienna, most notably Friedrich Hasenöhrl (1874–1915), Hans Thirring (1888–1976), and Schrödinger (figure 2).^12^

This intellectual period that had characterized Vienna in the late-nineteenth and early twentieth centuries was abruptly shattered by the annexation of Austria by Nazi Germany in 1938. Sharing the same regrettable fate of an enormous number of European intellectuals, around 50% of Viennese physicists were dismissed, and 40% of them forced to emigrate.^13^ After World War II, the academic culture underwent a substantial change: the long-standing symbiosis between physics and philosophy—that was characterized by the exceptional cultural golden age of pre-war Vienna—came to an end. As we shall see, however, in contrast to the ostensible scientific atmosphere, exemplified by the sentiment of “shut up and calculate,” in Vienna physics and philosophy maintained an underlying connection due to the continued interest of scientists that were influenced by the preceding philosophical golden age.

## The Postwar Era

### Reconstructing Physics in Vienna: Walter Thirring

Like for most other activities, reconstructing science in Austria after the Nazi period and the war required a great effort, and quite some time (it should also be recalled that Austria only regained sovereignty in 1955). Some of the physicists were reinstated, in particular Hans Thirring as the only professor of theoretical physics at the University of Vienna. However, his intellectual interests had changed somewhat. Immediately after the war, he started devoting a great deal of his interests to pacifist and political initiatives. Paul Feyerabend, who was a student of physics in Vienna in those years, recalled him saying: “This is important, physics is not.”^14^ Thirring became a pioneer of anti-nuclear movements, writing a book on the history of the atomic bomb,^15^ and serving as the only Austrian representative in the famous Pugwash Conferences on Science and World Affairs, an initiative initiated by the Russel–Einstein Manifesto against nuclear weapons.^16^ Some years later, Thirring also engaged in professional politics, serving as a member of the Austrian Federal Council (*Bundesrat*) for the Socialist Party of Austria (today Social Democratic Party of Austria, SPÖ) between 1957 and 1964.^17^ In that role, he promoted further pacifist initiatives, such as a proposed unilateral disarmament of Austria, known as the “Thirring Plan.”

Thirring’s deep involvement in complex political challenges, however, most likely came at the expense of his scientific achievements and the quality of his physics teaching. This was a potential reason that university courses in Vienna lagged behind the scientific developments of the time. Pietschmann, who had started his studies in physics at the University of Vienna in 1954, recalls that “it was kind of transition period. In those days there was no quantum physics taught at the University of Vienna.… Not at all!”^18^ The only other theoretician—a lecturer (*Dozent*) who was said to know quantum physics—was Theodor Sexl (1899–1967), who, however, also never taught it in his courses. A story circulated among the students at that time that he had written a book on quantum mechanics that was lost in the mail on its way to the publisher, and he therefore refused to teach quantum physics.^19^ Whether there is truth in this anecdote or otherwise, as a matter of fact, after World War II neither Hans Thirring nor Theodor Sexl, nor any other professor, taught quantum physics at the University of Vienna until as late as 1959.^20^ The students showed a great deal of initiative and used to meet in self-organized groups after the official lectures to study quantum physics using the famous book *Principles of Quantum Mechanics* by Paul Dirac.^21^

Only from the mid-1950s did the real postwar modernization of Viennese physics take hold. First, Berta Karlik (1904–90) was appointed professor of experimental nuclear physics in 1956, being the first woman in the history of Austria to become a full professor (*ordentliche Professorin*).^22^ Moreover, concerning fundamental physics, at the beginning of the same year, Schrödinger returned to Vienna as “honorary professor” (*Ehrenprofessor*), where he remained until his death in 1961.^23^ This surely changed the landscape of Viennese physics, causing great excitement among students, who, however, were to see some of their expectations betrayed. It is true that Schrödinger stimulated young physicists through his thought-provoking talks, in which foundational problems played a central role, such as his inaugural address on “The Crisis of the Atomic Concept,”^24^ and that he engaged in public philosophical debates, for instance when Victor Weisskopf (1908–2002) visited Vienna in 1958.^25^ Yet his impact on the Viennese research and education regarding foundational issues, especially those dealing with quantum mechanics, turns out to be much more limited.

First of all, during his years as a professor at the University of Vienna, Schrödinger mostly taught the course “General Relativity and Expanding Universes” (his very last lecture was held in March 1958),^26^ and only once around 1956 did he lecture on “wave mechanics.” Schrödinger’s awareness of being a dissident in his views on quantum theory persuaded him to not take any PhD students and thus prevented him from forming a school in Vienna. Pietschmann directly experienced this:

“[Schrödinger] said he could not take any graduate student because he was working only in two fields: one was general relativity and he simply had no good ideas. And then, you know, his eyes lightened with sparks coming out almost, and he said: ‘the other is wave mechanics. There, of course,’ he said, ‘I have lots of ideas.’ And then he said, and I kept this in mind: ‘I cannot take the responsibility to put a young man on a track which is considered to be a dead end by the rest of the world.’”^27^

Instead, perhaps the most remarkable change relevant to the modernization of physics in postwar Vienna stemmed from Thirring’s retirement and the stir caused by the search of a new professor of theoretical physics. According to Schrödinger’s biographer, “the various factions were agreed only on one thing: to prevent Schrödinger from using his enormous prestige to influence the decision.”^28^ In the end, quite shockingly, it was decided to appoint as the successor of Hans Thirring his son Walter Thirring (1927–2014). In Pietschmann’s words:

“there is one kind of nepotism which is worldwide unique, and this is Walter Thirring. I mean, no doubts about his capabilities, but he inherited the chair from his father. So Schrödinger was against it … because he said it is simply impossible to inherit a chair at the University.”^29^

After his PhD, Walter Thirring, most likely also thanks to the acquaintances of his father, went on a kind of Grand Tour to perfect his formation under some of the most distinguished living physicists of that time: he was in Dublin with Schrödinger, in Glasgow with Bruno Touschek, in Göttingen with Werner Heisenberg, in Zurich with Wolfgang Pauli, and in Princeton, where he had contacts with Einstein. When the younger Thirring finally came back to Vienna in 1959 as a full professor, he was an internationally recognized expert in quantum field theory.^30^

Thirring initiated modern physics in postwar Vienna. He was the first professor to teach quantum mechanics, together with advanced courses on contemporary physics. He also played a major role in making Austria a member state of CERN—where hedirected the Theory Division between 1968 and 1971—which helped put Austria back on the map for physics research at the international level. Moreover, Thirring attracted many young students who completed their dissertations with him, both on theoretical high energy physics and on general relativity. Many of that new generation of students (and the immediately following one) were to become professors at the University of Vienna and rebuild the physics landscape there. In particular, two of Thirring’s first students in Vienna, Herbert Pietschmann and Roman Ulrich Sexl (the nephew of the physicist Theodor Sexl), became professors at the end of the 1960s, and, as we shall see in the next section, helped restore the Viennese tradition of connecting physics with philosophy.

### Reconnecting Physics with Philosophy: Pietschmann and Sexl

Thirring himself did not show a particular interest in philosophical or interpretational issues, favoring formal problems of theoretical high energy physics: “He was very pragmatic,” recalls Peter Aichelburg, a former student of his who was to become professor of gravitational physics in Vienna.^31^ Nevertheless, Thirring was not only tolerant but, to a certain extent, even supportive of the initiatives that some of his collaborators and former students, foremost Pietschmann and Sexl, launched in the mid-1960s.

Subsequent to Schrödinger’s refusal to take him (or anyone else) as a student, Pietschmann completed his PhD in 1960 under Walter Thirring and became professor in Vienna in 1968 after collecting some international experiences. Only a few months later, however, Thirring left for CERN, and Pietschmann remained the only professor of theoretical physics in Vienna and became de facto head of the Theoretical Physical Institute. Pietschmann was always sensitive to the more speculative and philosophical aspect of theoretical physics. He had been introduced to the foundational problems of quantum mechanics in his years as a student, thanks to the presence of Schrödinger,^32^ but it was only in 1964 that Pietschmann—together with his friend, the philosopher Gerhard Schwarz—established a novel dialogue between physics and philosophy. The two founded the *Philosophisch-Naturwissenschaftlicher Arbeitskreis* (PNA), a discussion group that focused on topics at the intersection of physics and philosophy, debated in front of an audience of students.^33^ Since the beginning, Pietschmann and Schwarz wanted to establish the PNA as an official university course. They individually approached their respective supervisors for their approval. Pietschmann recalls that “Thirring always was very open minded and said: ‘yes, it is fine if I don’t have to be involved.’”^34^ From the side of the philosophers, the response was somewhat more pessimistic about the success of the course. Schwarz recalls his mentor saying: “this is not gonna work Mr. Schwarz, because physicists don’t understand the first thing about philosophy and it is not possible to teach them. But if you want to do that!”^35^ Despite this reluctance, the PNA took off as an official university course where students could get a grade by attending the panel discussions and handing in a paper.

Soon after its foundation, another prominent participant joined the PNA, Pietschmann’s colleague Roman U. Sexl. The latter was the nephew of the aforementioned Viennese physicist Theodor Sexl, from whom Roman possibly learned about the fundamental problems of quantum physics.^36^ Sexl completed his PhD in 1961 under Walther Thirring and, after a period at several US institutions, returned to Vienna in 1967, where he became full professor of general relativity theory and cosmology in 1971. His work on the production of particles by gravitational fields,^37^ carried out with his Viennese colleague Helmuth Urbantke, pioneered the field of particle production in curved spacetime that would lead to Stephen Hawking’s radiation theory a few years later, and won him international fame. Moreover, Sexl became renowned for his involvement in physics education and wrote several scholarly and popular science books. Sexl had a dynamic intellectual style. As Bertlmann recalled: “he was very broad in this. He was typically Viennese in this. Not just doing calculations, but also philosophy, culture, art.”^38^ Indeed, since 1979, Sexl was the editor of the book series *Facetten der Physik* (“Facets of Physics”), which promoted a genuinely interdisciplinary and open-minded approach to physics, as stated by the introductory editorial note that opens every volume of the series: “Physics has many facets: historical, technical, social, cultural, philosophical and amusing ones. They can serve as essential and decisive motives for the pursuit of the natural sciences.”

In that book series, Franco Selleri, the main character of the Italian revival of FQM in the 1970s (see below), published his *Die Debatte um die Quantentheorie* (“The Debate over the Quantum Theory”) in 1983.^39^ Sexl himself—together with Kurt Baumann—published, in 1984, *Die Deutungen der Quantentheorie* (“The Interpretations of Quantum Theory”),^40^ a collection of original papers that aims to show different interpretations of quantum formalism (they include papers by the Copenhagen school, as well as the alternative views of Schrödinger, Fock, Bohm, Bell and deWitt). Remarkably, Sexl’s book shows an exceptionally modern understanding of the relevance of the interpretational problems:

“Because only mathematical formalisms that have an interpretation can be understood as a physical theory.… Even though, at some point in time, different interpretations lead to the same physical results, they let us anticipate very different developments and research directions.”^41^

Moreover, together with Peter Aichelburg, Sexl edited a collective volume in honor of Einstein on the occasion of the centenary of his birth. The book, *Albert Einstein: His Influence on Physics, Philosophy and Politics*,^42^ collected contributions from distinguished physicists and philosophers.^43^ It also contained a chapter by Nathan Rosen on the EPR paradox, wherein Einstein’s collaborator stated his lack of confidence that quantum theory could be completed in terms of hidden variables, because this would mean to give up locality in the light of Bell’s theorem.

In the spirit of *Facets of Physics*, the PNA covered a diverse palette of topics in their discussions, each of which usually took up several semesters. In the course of over fifty years, the PNA covered twelve broad topics in their discussions.^44^ It started with the subject of space and time (1964–69), which was to become a common theme over the course of the following decades. Its continuation led to questions regarding inertial and gravitational mass (1969–71), where the assumptions leading to ideas such as cosmic inflation and the theory of the big bang were received critically by the philosophers of the PNA, who considered them rather arbitrary. This lead Pietschmann to introduce the distinction between predictive and descriptive theories.^45^ As a consequence, the discussion shifted toward methodology (*Methodenproblem*) (1971–78).

In their meetings, they sometimes invited guests to contribute to the discussion, such as Ingvild Birkhan, Carl Friedrich von Weizsäcker, and Roland Fischer. Of particular interest is the involvement of Karl Popper, who became a major influence on Pietschmann’s thoughts. Sexl and Pietschmann invited Popper to participate in the discussion of the PNA in October 1972, and kept a correspondence with him until 1980.^46^ Pietschmann published the paper “The Rules of Scientific Discovery Demonstrated from Examples of the Physics of Elementary Particles,” wherein he showed that the methodological rules of Popper’s falsificationism are “actual tools rather than abstract norms in the development of physics.”^47^ Despite his influence on the PNA and the repeated invitations, there is no evidence that Popper ever attended their meetings.

The PNA certainly had a strong impact on the general landscape of physics in postwar Vienna, where the new developments of high energy physics, under the lead of Walter Thirring, had overcome the philosophy-oriented mood that had characterized prewar physics research. In Pietschmann’s case, for example, the interaction with philosophers was to have lasting and deep consequences, and paved the way for his subsequent interest in foundational questions. This is witnessed by Aichelburg, who did not find the PNA particularly fruitful, but recalls that “Pietschmann … somehow absorbed this philosophical vocabulary and somehow turned to the philosophical aspects.”^48^ Zeilinger also participated in at least one of the meetings of the PNA, but did not find it particular influential in his intellectual development. However, he maintains that the very existence of such a successful initiative was symptomatic of a remarkable openness to foundational questions in the Viennese environment, which likely helped pave the way for the establishment of FQM in the following years.^49^ The PNA was so successful as to last for over half a century. A final meeting of the PNA took place in 2016.

Before entering the core of our reconstruction of the first steps toward modern FQM in Vienna, however, we deem it relevant to give an overview of the system of physics education at the University of Vienna in the 1960s and 1970s. As mentioned in the introduction, a peculiar aspect of the University of Vienna is that up until 1975 the physics institutes were part of the Faculty of Philosophy. This was not just an administrative matter, but had a real impact on the education of young physicists. Pietschmann recalls that physics students “had to take five *Rigorosen*, [final exams for a doctorate] three in the main topic—in [this] case physics—and two in philosophy. So [they] really had to study it and learn it, although the philosophers knew that they had to be very soft with scientists.”^50^

Zeilinger also recalls that the course he followed in philosophy to prepare for the *Rigorosen* had an impact on his intellectual formation.^51^ In general, this formal training in philosophy is likely to have played a considerable role in forming the interest in foundational problems at Vienna.

Moreover, the curriculum was exceptionally flexible, granting students customized training. Bertlmann remembers: “When I think back it was like in paradise, I must say. You only had to choose your thesis advisor and he told you what you had to study.”^52^ Indeed, until the reform of 1975, there was a single cycle degree program (which if completed would grant a PhD) without mandatory courses or exams to take, except the final *Rigorosen*. Zeilinger, who studied physics in Vienna in the same years as Bertlmann, recalls:

“when I started to study physics and mathematics at the University of Vienna in 1963, there was no fixed curriculum at all. One was essentially free to choose the topics according to one’s liking. Only at the end, one had to pass a rigorous examination and present a PhD thesis. This resulted in me taking not even a single hour of quantum mechanics, but I learned it all from textbooks for the final exam.”^53^

The first course of quantum mechanics was taught by Walter Thirring in 1959. However, it did not cover any foundational problems, such as the EPR paradox or the measurement problem in quantum mechanics. During the years 1968–1971, when Thirring moved to CERN, Pietschmann replaced him teaching theoretical physics, a two-year course divided in four parts: mechanics, electrodynamics, quantum mechanics, and statistical physics.^54^ However, despite his full involvement in philosophical discussions, topics pertaining to FQM were not introduced in this course. The same lack of foundational topics is apparent in the courses led by Sexl, who also taught quantum mechanics in the following years. Yet, despite the fact that *Facetten der Physik* was publishing whole volumes on FQM, and their involvement in the PNA, neither Pietschmann nor Sexl seemed to have introduced their interest in foundational problems into their teaching throughout the 1970s and even the 1980s. Bertlmann, who attended their courses, recalls:

“The lectures of Pietschmann and Sexl were very good, very modern, but no density matrices and of course, no Bell’s Theorem. Although Sexl was writing about Bell’s Theorem in a book at that time, so he knew about it. But he never mentioned it in the course. It was an official line not to do it.”^55^

One can thus see a dichotomy between the interest in foundational and philosophical aspects of physics that physicists such as Pietschamann and Sexl were extensively cultivating and their appearance as professors of the Faculty of Physics, especially in their teaching. Aichelburg maintains: “if there was at that time some discussions on fundamental physics, I didn’t know this.”^56^ We regard this attitude of keeping these interests somewhat private as a cultural product of the age of “shut up and calculate.” As Bertlmann puts it, “it was an official line.”^57^

## The Rise of Quantum Foundations in Vienna

### Experimental FQM with Neutrons: Rauch and Zeilinger

The *Atominstitut*—an inter-university institution between the Technical University of Vienna (TU) and the University of Vienna—was a major venue for the rebirth of the Viennese foundational research. Helmut Rauch, one of the protagonists of the rise of FQM in Vienna was based there. During his study years at TU, “quantum mechanics was taught in the standard form, that means Copenhagen as it is, and there were no broad discussions about that.”^58^ TU never had a Faculty of Philosophy, which might explain why the connections to philosophy were weaker then at Vienna. However, one can find some exceptions: Walter Glaser (1906–60), professor of Theoretical Physics at TU since 1956, was said to have strong interest in FQM, and to oppose the Copenhagen interpretation. Furthermore, Rauch recalled that occasional visitors would come to the TU to give talks about Bohmian mechanics, and that Karl Popper visited the institute several times around the mid-1960s, because he was very interested in matter-wave quantum phenomena (see below).^59^

Rauch’s doctoral supervisor, Gustav Ortner (1900–1984)^60^ “was very open and … interested of doing fundamental physics which could be published in reasonable journals.”^61^ In 1972, Rauch was appointed full professor of experimental and neutron physics at TU, and in the following years he was to be recognized as one of the most renowned experts on neutron interferometry worldwide. Indeed Rauch’s group obtained a pivotal result in 1974, becoming the first to realize a silicon crystal appropriately manufactured to implement a (Laue-type) interferometer between coherent beams of neutrons.^62^

In 1968, Anton Zeilinger—at that time a student at the University of Vienna—also joined Rauch’s group, wherein, in 1971, he completed his doctoral dissertation. Zeilinger recalls that his interest on foundations of physics was easily accepted and grew within Rauch’s group at the Atominstitut, feeling that he had escaped the reluctance with which foundational issues were still regarded at the Vienna Faculty of Physics.^63^ Zeilinger was particularly involved in the first experiment of foundational nature conducted with the new neutron interferometer: the verification of the sign flip of the wave function of a spin-1/2 particle.^64^ This seminal result was a turning point in Zeilinger’s career because this offered him the opportunity to get involved with the (at that time still rather small and unconventional) community of FQM. In fact, Zeilinger participated in an international workshop on FQM, “Thinkshop on Physics,” which took place in Erice (Italy) in 1976 and was directed by John Bell and Bernard D’Espagnat (1921–2015).^65^ This workshop had a pivotal international resonance for the field of FQM, as it “was seen by some physicists as the turning point in the acceptance that quantum nonlocality was indeed a new physical effect.”^66^ John Clauser (b. 1942), a pioneer of research on Bell’s inequalities, pointed out that “the sociology of the conference was as interesting as was its physics. The quantum subculture finally had come out of the closet.”^67^ There, Zeilinger had his first encounter with quantum entanglement and Bell’s inequalities:

“[T]his was my first real encounter with the international scientific community. There, I heard for the first time about Bell’s theorem, about the Einstein-Podolsky-Rosen paradox, about entanglement, and the like.… This meeting turned out to be very crucial in my life. There, I met a number of colleagues for the first time, some of whom later became personal friends.”^68^

It should be stressed that Zeilinger’s research did not deal with entanglement and non-locality until 1985, when, on the occasion of the fiftieth anniversary of the Einstein-Podolski-Rosen (EPR) paper, a conference was held in Joensuu (Finland). For this occasion Zeilinger and Michael Horne (1943–2019) formulated the first experimental proposal for violating Bell’s inequalities using entanglement between external degrees of freedom (as opposed to internal ones, such as spin).^69^ That work, published in the proceedings of the conference, was the first paper of Zeilinger on matters of entanglement and non-locality.^70^ Zeilinger and Horn’s collaboration continued in the following years and was joined by Daniel Greenberger (b. 1932) in 1987, with whom they were the first to tackle the problem of tripartite entanglement, allowing them to formulate a stronger form of Bell’s theorem—the GHZ theorem.^71^

Zeilinger was predominantly an experimentalist, so his aim became to realize some of these theoretical proposals involving multipartite entanglement in a laboratory. However, he soon realized that neutrons are not suitable for creating entangled states, so he switched to photonic entanglement, applying the newly developed techniques for creating entangled pairs of photons through the effect called spontaneous parametric down-conversion.

Zeilinger became increasingly recognized as a world’s leading figure in FQM, quantum optics, and quantum communication. Reconstructing Zeilinger’s scientific achievements in these fields goes beyond the scope of the present paper.^72^

But it is worth noting that, on Zeilinger’s (amongst others’) initiative, the “Institute for Quantum Optics and Quantum Information” (IQOQI) of the Austrian Academy of Sciences was founded in 2003, with two independent sites in Innsbruck and Vienna.^73^

### The Influence of the “Quantum Dissidents” on Vienna’s FQM

As already noted, FQM encountered a tremendous setback in the postwar period, with the exception of a few physicists that historian of physics Olival Freire Jr. has named the “quantum dissidents” because they challenged the standard view according to which “foundational issues were already solved by the founding fathers of quantum physics.”^74^ It ought to be remarked that the majority of these dissidents—such as David Bohm (1917–92), John Bell, Hugh Everett (1930–82), Jean-Pierre Vigier (1920–2004), and Franco Selleri—were motivated by realism to oppose the widespread Copenhagen interpretation of quantum mechanics. On the other hand, the school of thought initiated by Zeilinger in Vienna, coming from a quantum information background, mainly supports a non-realist “neo-Copenhagen” interpretation, where the epistemic concept of information plays a major role.^75^

Nevertheless, some of the aforementioned quantum dissidents helped popularize FQM in Vienna and have likely played a relevant role in smoothing the path toward the rise of the “Zeilinger era” of FQM.

The initiator and main actor of the rebirth of the interest in FQM in Italy was the theoretician Franco Selleri.^76^ Selleri’s realist position was explicitly rooted in his radical left-wing, materialistic credo. In the 1970s, a remarkably large group of young physicists—who included Giancarlo Ghirardi (1935–2018), who would make important contributions to FQM—joined his research program.

Selleri and his school took part in the Erice workshop of 1976, and more than likely it is there that he started to be interested in experimental tests of some of his ideas on FQM using neutrons. Many years later, he would recall: “I have done a lot to promote neutron interferometry as a tool to study fundamental questions.… I was particularly connected with Helmut Rauch at Vienna and Rauch is a practical man, less philosophically inclined than Zeilinger but a very concrete physicist. And I believe that neutron interferometry shows very clearly that you have wave particle duality again.”^77^

Indeed, after 1982 when the first experiments on Bell’s inequality were conducted with photons by Alain Aspect (b. 1947)^78^—which confirmed the violation predicted by quantum theory and thus jeopardized most of the hidden variable programs—neutron interferometry became for many the hope to save hidden variables. John Bell himself explained to Rauch that “it would be important to make it [Bell’s experiment] with matter waves, because electromagnetic waves are in principle all over.”^79^ This view was shared by French theoretical physicist Jean-Pierre Vigier, who visited Rauch’s group several times, both at the Atominstitut in Vienna and at the Institut Laue–Langevin in Grenoble, where he tried to persuade Rauch to conduct experiments that could allegedly disprove the Copenhagen interpretation.^80^ Indeed, Rauch stated:

“many of our experiments were afterwards really stimulated by some strange ideas about how to distinguish between different interpretations.… Also Vigier was a fighter for that. But … we were fortunately always very careful in our discussion. We never mentioned that it disproves quantum mechanics.”^81^

In the early 1980s, Vigier was the main influence on the octogenarian philosopher Karl Popper—who had been engaging with problems of FQM since 1934 and as such should be considered a fully-fledged “quantum dissident”^82^—having actively re-engaged with the community of quantum physicists. At a conference in Bari, organized by Selleri himself, Popper presented a “simplified version of the EPR experiment.” Selleri had arranged that some experimentalists, including Rauch, would be present with the hope of persuading someone to perform Popper’s proposed experiment.^83^ Consequently, Popper visited Rauch in Vienna in March 1984. Rauch wrote to Popper that he found his proposal very interesting and invited him to speak at the *Chemisch-Physikalische Gesellschaft* (“Chemical-Physical Society”) in Vienna, of which Rauch was the chairman.^84^ Popper’s experiment was carried out only in 1999, after his death. It turned out that Popper’s proposal was not capable of discriminating between different interpretations of quantum physics.^85^ On the other hand, Bell’s experiments with neutrons did not work because of the technical difficulties with realizing a source of entangled neutrons.^86^ However, the interaction between these unorthodox thinkers and the group of Rauch stimulated the foundational character of the experiments with neutrons, as explicitly acknowledged by Rauch, and more generally may have contributed to open to discussions on FQM in Vienna.

Moreover, Selleri had been good friends with Pietschmann for many years (they met at CERN in 1960–61 when Selleri was still working on particle physics) and, from the beginning of the 1980s, began a fruitful intellectual relationship with Sexl too. It was most likely Sexl and Pietschmann’s interest in foundational questions that caused Selleri to spend a leave of absence at the institute of physics in Vienna. He visited from winter 1980 to spring 1981 and made further regular visits until at least 1985.^87^ In Vienna, indeed, Selleri discussed FQM mainly with Pietschmann, Sexl, and Rauch. Bertlmann, still a young researcher at that time, also remembers Selleri’s visits, but gives us a taste of how unpopular the topic of FQM was: Bertlmann “did not have contact with him [Selleri] on this subject. This was not allowed, so to say.”^88^ Therefore, it is surprising that, during his period in Vienna, Selleri was actually allowed to give lectures on FQM. Selleri himself remembered that Sexl “followed [his] lectures in Vienna and invited [him] to write a book” on their topic,^89^ which appeared in 1983 as a volume in the already mentioned series *Facetten der Physik*, edited by Sexl himself (see above).^90^ The contents of this book reflect the unconventional palette of topics that Selleri had covered in his lectures in 1980. It encompasses whole chapters devoted to problems such as the completeness of quantum theory and hidden variables, wave-particle duality from a realist point of view (which includes also experiments on neutron interferometry), the Einstein-Podolsky-Rosen thought experiment and Bell’s inequality, as well as a final chapter on “Experimental Philosophy.”

Moreover, while in Vienna, Selleri wrote a paper^91^ in which he proposed a model that allegedly disproved the fact that the factorability of probabilities for outcomes of experiments conducted at distant locations is sufficient to derive Bell’s inequalities, as proposed by Clauser and Horne.^92^ This proposal earned the praise of Popper—who coined the term “universality claim” to refer to the result of Clauser and Horne^93^—and had a certain following throughout the 1980s. However, it turned out to be untenable because it was based on a misconception.^94^

Selleri is also known for having been among the first proposers of protocols to achieve superluminal communication, based on (a misconception of) quantum entanglement.^95^ He had proposed this independently of the works of Nick Herbert (b. 1936), who had promoted the same idea in the United States.^96^ It was in Vienna that Selleri explored one such scheme for achieving faster-than-light communication. This, however, assumed the possibility of a laser emitting longitudinal modes all with the same, yet unknown, circular polarization. Pietschmann remembers that Selleri entered his office jubilantly and presented this result to him and Walter Thirring. The proposal had no formal mistakes, but Pietschmann replied: “Absolutely great, you have just proven that it is impossible to make a laser which lases linearly polarised light in the same way,” and Thirring agreed.^97^ Indeed, the impossibility of preparing copies of unknown quantum states was to be proven in full generality soon afterwards, motivated also as a reaction to Nick Herbert’s proposal,^98^ and became known as the “no-cloning theorem,” on which the entire field of modern quantum cryptography relies.

Interestingly, Selleri and Zeilinger co-authored a paper in 1988,^99^ despite their opposite views on FQM. Therein, they analyzed a local deterministic hidden variable model that would determine whether a photon is detected or not in an EPR-like experiment. This model is empirically distinguishable from quantum mechanics, but this was at that time out of experimental reach, due to the low efficiency of photon detectors. The disagreement between the two authors is made manifest in the last sentence of the paper: “the difference in the expectations of the present authors whether this will happen or not is indicative of the diversity of opinion among physicists at large.”^100^ On this note, Zeilinger recently recalls: “I saw the paper as a mathematical exercise out of curiosity, for Selleri it was important for his realist view.”^101^ As a matter of fact, all experiments so far performed confirm quantum mechanics.

The quantum dissident *par excellence*, the physicist who changed the understanding of modern FQM more than anyone, was John Bell. He also played a role in Vienna, mostly indirectly through the Austrian physicist Reinhold Bertlmann.^102^ The latter completed his dissertation in 1974 under Pietschmann’s supervision. In these years, Bertlmann was active in left-wing student movements, which were particularly sensitized to physicists’ involvement in military research (and more specifically in the Vietnam War). Bertlmann’s leftist attitude led to a curious wearing habit that was to change his scientific life some years later. Bertlmann recollects:

“my way of looking was a protest against the bourgeoisie, but not only this. I don’t know how it actually happened.… One day I changed my socks. I thought: ‘why do all people choose the socks of the same color for both feet, why is this?’ So this for me was like a sheep-effect. And then I changed this.… I don’t know the day exactly when I started, must have been in the ‘60 s, but from that day on I never had socks of the same color, no day.”^103^

In 1977, Bertlmann had the opportunity to work for nine months in the Soviet Union, at the Joint Institute for Nuclear Research in Dubna. It should be recalled that Austria declared itself “perpetually neutral” in its new Constitution of 1955 (hence, it did not belong to either NATO or the Warsaw Pact), so it enjoyed a status that allowed scientific collaborations to develop across the Iron Curtain. There, Bertlmann was exposed for the first time to alternative interpretations of quantum mechanics, in particular to the realist-materialist position of Dmitrii Blokhintsev (1908–79) who had written a renowned book (within the Soviet Union) on FQM.^104^

In April 1978, Bertlmann moved to the Theory Division of CERN, where he soon made Bell’s acquaintance.^105^ They started a close friendship and a prolific collaboration on topics of particle physics.^106^ Bell was a recognized authority in theoretical particle physics and accelerator physics—he was called “the Oracle of CERN”^107^—but he never discussed his paramount results on FQM (Bell’s inequalities had been published as early as 1964!)^108^ with his colleagues in CERN, nor did he discuss it with Bertlmann, who recalls: “John … never mentioned his quantum works to me in the first years of our collaboration. Why? This I understood later on, John was reluctant to push somebody into a field that was quite unpopular at that time.”^109^

The first direct contact between Bell and the University of Vienna occurred in May 1980, when he was invited by Walter Thirring as the “Schrödinger Guest Professor” for about ten days. On that occasion, Bell gave three lectures. It must have been quite for Thirring to figure out that only the first one was about particle physics, “On the role of duality for bound states in QCD” (on May 19), whereas the other two focused on his (at that time) virtually unknown work on FQM, “Assuming the Schrödinger equation is exact” (on May 21), and “On locality in quantum mechanics” (on May 22).^110^ During the last talk, Bell joked that EPR pairs behave in the same fashion as Bertlmann’s socks. This triggered the laughs of the audience, but nobody, including Bertlmann, thought more about it.^111^ However, in 1980, the joke came back into Bertlmann’s life, launching him, willing or not, “out of the blue into the middle of the quantum debate,”^112^ when Bell published the paper “Bertlmann’s socks and the nature of reality.”^113^ Therein he explained the fundamental difference between “spooky” quantum correlations and classical ones, exemplified by the (anti-)correlated colors of Bertlmann’s socks. Indeed, the paper begins with:

The philosopher in the street, who has not suffered a course in quantum mechanics, is quite unimpressed by Einstein-Podolsky-Rosen correlations. He can point to many examples of similar correlations in every day life. The case of Bertlmann’s socks is often cited. Dr. Bertlmann likes to wear two socks of different colours. Which colour he will have on a given foot on a given day is quite unpredictable. But when you see (Fig. 12) that the first sock is pink you can be already sure that the second sock will not be pink.… And is not the EPR business just the same?”^114^

Bertlmann recalls that the paper pushed him abruptly into the field of FQM: “I had to study because everyone was addressing me as the most expert in the world, and I knew nothing!”^115^ And so he did, engaging in many discussion on FQM with Bell himself. Together with Jun John Sakurai (1933–82), who was at CERN at the beginning of the 1980s—until his untimely death—he wrote his famous book on quantum mechanics, which was the first manual for students to include Bell’s inequalities.^116^ Indeed, throughout the 1980s, Bertlmann became a main actor in popularizing Bell’s results on FQM both in Vienna and internationally. He started being invited to give talks about Bell’s inequalities and quantum non-locality, especially by physics groups that never dealt with FQM. In 1984, invited by Othmar Preining (1927–2007) at the Institute of Experimental Physics in Vienna, he gave the talk “*Bell’sches Theorem*” (“Bell’s theorem”); in 1987 he presented the lecture “Bell’s theorem and the nature of reality” first again at the University of Vienna, and in the same year—invited respectively by Heinrich Leutwyler (b. 1938) and Hans Günter Dosch (b. 1936)—in Bern and in Heidelberg. Moreover, at the end of 1986, Bertlmann persuaded Walter Thirring to organize a conference on FQM at the University of Vienna, with an emphasis on Bell’s Theorem. The “Schrödinger Symposium” took place the following year and, naturally, Bell was invited as a speaker. He spoke on “Schrödinger’s cat” (on September 17, 1987), and took part in the panel discussion (together with Zeilinger), announcing therein his notorious list of “words that should be forbidden in serious discussion,” such as “system,” “apparatus,” “observable,” and “measurement.”^117^

Throughout the 1980s—mainly thanks to experimental violations of Bell’s inequalities, but also thanks to the popularization of this topic by the quantum dissidents and other younger scholars—Bell’s theorem became increasingly recognized as proper physics. On the occasion of Bell’s sixtieth birthday, in 1988, Bertlmann wrote the paper “Bell’s theorem and the nature of reality,”^118^ the pre-print of which he sent to Bell, who enjoyed it very much,^119^ but also to Abner Shimony (1928–2015), another of the early experts in Bell’s theorem. The latter, who involved James Cushing (1937–2002), replied enthusiastically with the proposal of organizing a volume of invited papers dedicated to Bell’s work on FQM. With the help of the editor of the journal *Foundations of Physics*, Alwyn Van der Merwe (1927–), they started inviting some of the most prominent physicists and philosophers with the aim of giving long-overdue credit to Bell’s work on FQM. Bell was unaware of this project, orchestrated behind his back to surprise him. Writing the papers and assembling the volume, however, took two years and the first dedicated issue appeared on October 1, 1990^120^. Most sadly, Bell died unexpectedly that very day, without knowing of the honor that he was about to receive.^121^

### Foundations of Quantum Mechanics “Come out of the Closet,” Also in Vienna

Interest in FQM took off shortly after Bell’s death in the 1990s, with the advent of quantum information theory and applications for quantum communication.^122^ This was also the case in Vienna.

Curiously enough, although Bertlmann and Zeilinger were exactly the same age, both studied at the University of Vienna, and both had a common interest in FQM (at least throughout the 1980s), they did not get to know each other until 1991. The occasion was provided by a conference in memory of John Bell, “Bell’s theorem and the foundations of modern physics,” organized by Selleri and collaborators on October 7–10, in Cesena, Italy. Bertlmann recalls: “There I met [Zeilinger], we found common interests and we thought, let’s begin to do something. And we had this idea: let’s educate young people and with this young people we can do something. Then we thought the best could be to organize a joint seminar.”^123^ In 1994, they established the seminar “Foundations of Quantum Mechanics” as an official course between the University of Vienna, where Bertlmann was associate professor, and the University of Innsbruck, where Zeilinger had just become full professor. This was activated as an official course thanks to the support of Pietschmann, in his role of head of the Physics Institute.

The seminar, which had a “quite informal, familiar character,”^124^ turned out to be a turning point in the acceptance of FQM at the University of Vienna, for it sensitized many students to foundational problems that were never discussed in official courses. Shortly afterwards, the first Diploma theses (the equivalent of a master’s level) on FQM started appearing: Časlav Brukner (b. 1967) who was to become full professor of quantum information theory and foundations of quantum physics at the University of Vienna—was the first to complete his thesis in 1995 at the University of Vienna under Zeilinger (who was still in Innsbruck) and Pietschmann. In 1997, Dominique Groß with the thesis “*Die Nichtlokalität in der Quantenmechanik*” (“Nonlocality in Quantum Mechanics”), supervised by Bertlmann, was the first student to have directly been influenced by the quantum foundation seminar. Beginning in 1996, Bertlmann also modernized the way quantum mechanics was taught in basic undergraduate courses, discussing “what a wave-function means, what is a wave-function collapse” and introducing the density matrix formalism and Bell’s inequalities.^125^

Besides Zeilinger’s research activities at the *Atominstitut* and at the Institute for Theoretical Physics at TU, a few more people started doing research on FQM such as Johannes Summhammer, Günter Krenn (in collaboration with Zeilinger), and Karl Svozil.^126^ Summhammer published one of the first partial reconstructions of quantum theory, deriving Malus’s law from first principles,^127^ and Svozil carried out extensive research on quantum contextuality.^128^ Bertlmann, too, finally entered active research in FQM in the mid-1990s, together with Walter Grimus (b. 1953), a professor of theoretical particle physics at the University of Vienna. They investigated the possibility of performing Bell’s experiments with massive particles, such as *B*-meson pairs, which are naturally produced in entangled states in accelerators due to conservation laws. Their collaboration lasted about a decade, and included several of their students and collaborators.^129^

When Zeilinger came back to Vienna in 1999 (this time to stay) together with Bertlmann they organized a big conference on FQM, “Quantum [Un]Speakables.” The conference took place at the University of Vienna on November 10–14, 2000, commemorating the tenth anniversary of Bell’s death, and gathered the most distinguished scholars working in FQM and quantum information (and a few from particle physics). Symbolically taking place at the turn of the new century, this conference marked the acceptance of FQM into the domain of physics in Vienna. Walter Thirring, who more than anyone else personified the renaissance of modern physics after World War II, came to realize his underappreciation of Bell’s results in FQM by pronouncing: “I have to apologize to John Bell, that I recognized the significance of Bell’s theorem only so late.”^130^

## FQM in Today’s Vienna

Since the 2000s, Vienna has seen exceptional growth of quantum information science and quantum technology, all branches of physics stemming more or less directly from FQM. Zeilinger, who is now Emeritus Professor at University of Vienna, is today recognized among the world’s leading physicists for his work in the field of FQM. As of the date of publication, the Faculty of Physics of the University of Vienna has five full professors and three assistant professors, most of whom deal with either theoretical or experimental problems of FQM; the same is true at the IQOQI-Vienna which encompasses another six research groups, not counting the overlaps with the Faculty. These two institutions together amount to the impressive total number of roughly 150 researchers (including PhD students) working in this field.

## Conclusion

We have shown that today’s outstanding research landscape on FQM in Vienna did not come out of the blue by means of a single physicist (or a small group thereof), but rather developed through a long tradition of interest in philosophical and foundational problems. We have discussed the reconstruction of physics in post–World War II Vienna. In particular, we have elucidated the roles of Walter Thirring, who helped bring back Vienna on the international landscape of physics, and of the PNA, founded by Herbert Pietschmann and joined by Roman U. Sexl, which reintroduced the discourse between philosophy and physics to the Faculty of Physics. We have shown how modern developments unfolded and how Vienna became a center of foundations of physics, highlighting the roles of, among others, Helmuth Rauch, Anton Zeilinger, Reinhold Bertlmann, as well as external influences from the likes of Franco Selleri, Jean-Pierre Vigier, Karl Popper, and John Bell.

Since our research mainly relies on interviews and the analysis of significant social networks, it allows us to reconstruct otherwise inaccessible information about historical developments with a focus on the perception of the general atmosphere and certain lines of research. However, one should keep in mind that the subjective nature of our sources limits the reliability of our narrative up to certain extent, which is a well-known problem of oral history.^131^

In conclusion, the philosophical inclination of generations of Viennese physicists, as well as the initiatives they undertook, although those often remained hidden from the public appearance, played a crucial role in opening room for the establishment of the present-day thriving environment of foundational research in Vienna.
